# Clinical study of renal artery cold perfusion combined with laparoscopic nephron retention in the treatment of complex renal angiomyolipoma

**DOI:** 10.3389/fonc.2023.1220380

**Published:** 2023-10-18

**Authors:** ChaoShuai Zhu, HuaQi Yin, ShiMing Zhao, YongKang Ma, ZhengHui Sun, MingKai Zhu, Zheng Du, Tiejun Yang

**Affiliations:** Department of Urology, The Affiliated Cancer Hospital of Zhengzhou University, Zhengzhou University, Zhengzhou, China

**Keywords:** renal artery cold perfusion, laparoscopic nephron preservation, renal angiomyolipoma, GFR, renal artery balloon catheterization

## Abstract

**Objective:**

The aim of this study is to summarize the surgical experience of renal artery cold perfusion combined with laparoscopic nephron preserving surgery for the treatment of complex renal angiomyolipoma and to evaluate the safety and feasibility of this surgical protocol.

**Materials and methods:**

Clinical data of nine patients who received renal artery cold perfusion combined with laparoscopic nephron preserving surgery for complex renal angiomyolipoma in our hospital from February 2017 to August 2020 were retrospectively analyzed. The study parameters included imaging findings, total renal function before and after surgery, glomerular filtration rate (GFR) of affected kidney before and after surgery, and related complications.

**Results:**

Eight of the nine patients successfully completed the operation, one patient was intolerant to renal artery balloon implantation, and the success rate of the operation was 88.89%. The mean maximum tumor diameter was 6.8 cm, and RENAL score was 7 points. Postoperative total renal function and GFR of the affected kidney had no significant changes compared with that before surgery, and imaging examination showed no tumor residue or recurrence

**Conclusion:**

This surgical procedure is safe and feasible for complex renal angiomyolipoma and can be used as a surgical option for renal hamartoma. The long-term effect needs to be confirmed by further studies.

## Introduction

Renal hamartoma [also known as renal angiomyolipoma (RAML)] is a common benign tumor of the kidney, with an incidence of about 0.4% in the general population, accounting for 3% of all renal tumors (1). Treatment of RAML depends on its size, and studies have suggested that renal AML larger than 4 cm has a significantly higher risk of rupture and bleeding. Therefore, therapeutic inter vention is recommended for renal hamartoma with tumor diameter larger than 4 cm (2, 3). Currently, major surgical treatments for RAML include selective arterial embolization (SAE), nephron sparing surgery (NSS), and ablation (4). On the basis of a large number of retrospective studies on SAE and NSS, NSS has the advantages of complete tumor resection and low recurrence rate. Therefore, NSS may be a better choice for patients with RAML (5). However, to reduce intraoperative bleeding, it is inevitable to clamp the renal artery to block the blood supply to the kidney, which will aggravate the damage to the kidney function. Studies have shown that hot ischemia (>25–30 min) may also cause irreversible ischemic injury to surgically treated kidneys (6). An animal experimental study has confirmed that there is no absolutely safe time for renal hot ischemia. Experimental animals with hot ischemia for 26 min or more have serious renal injury, and renal pathology and serum creatinine (SCr) have significant changes. Heat ischemia for 18 to ~26 min caused mild kidney injury, pathological changes, and kidney molecular expression, but no changes in SCr (7), whereas cryogenic arterial cold perfusion can be applied to acute stroke, which is a feasible and promising tissue-protective therapy (8). Therefore, it is of great significance to study the surgical program of cryogenic arterial cold perfusion combined with laparoscopic nephron preservation surgery for reducing the incidence of postoperative renal injury, intraoperative bleeding, and postoperative complications in patients with hamartoma.

## Data and methods

**Figure 1:**
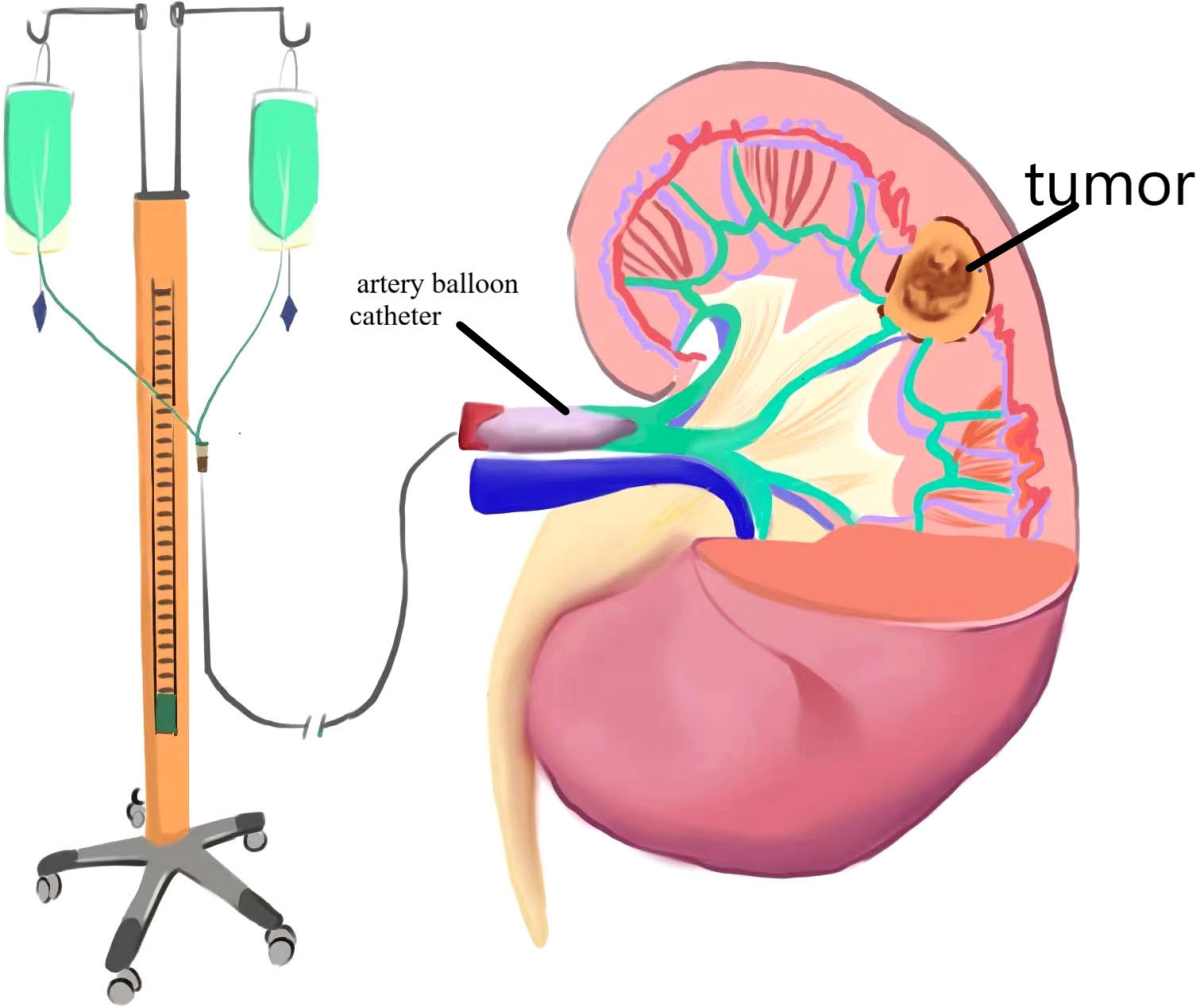
Renal artery intubation perfusion diagram.

**Figure 2:**
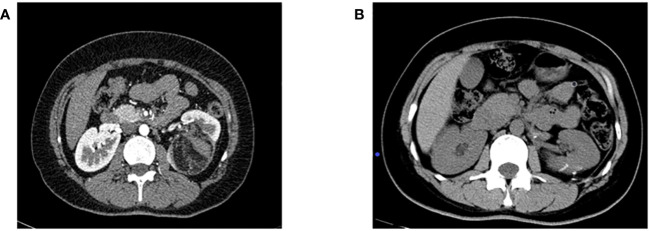
**(A)** Preoperative enhanced CT imaging results; **(B)** CT results of the patient 6 months after surgery.

1. Clinical data: From February 2017 to August 2020, nine patients with RAML were treated by renal artery cryogenic cold perfusion combined with laparoscopic nephron preserving surgery in our hospital, as reported below. Among the nine patients, there were two men and seven women, ranging in age from 24 to 52 years old, with an average age of 38.2 years old, three on the left side and six on the right side. Among them, five patients came to hospital with abdominal pain, and the remaining four patients had no obvious clinical symptoms. The maximum tumor diameter was 3.8–11.3 cm, and the average was 6.8 cm. RENAL scores ranged from 5 to 11, with a mean of 7. All patients under went enhanced CT or MRI before surgery.
2. Surgical method (right renal tumor as an example): 1. Renal artery balloon catheterization: The skin in the right groin area was disinfected, sterile sheet was applied, and the puncture site of the right femoral artery was given local anesthesia with 5 mL of 2% lidocaine. Seldinger puncture of the right femoral artery was performed. After successful insertion of 6F arterial catheter sheath, 5F snake catheter was inserted through the sheath into the affected renal artery for arteriography, and exchange guide wire was sent along the angiography catheter of the renal artery. The balloon was sent to the main stem of the affected renal artery along the exchange guide wire, and 2 mL of normal saline was injected into the balloon. The blood flow of the affected renal artery was significantly reduced, and the renal artery blood flow blocking effect was good. The liquid in the balloon was drained, the switch was turned off, and the balloon tube and vascular sheath *in vitro* were fixed. The schematic diagram of the operation is shown in **Figures 1**, **2**. Laparoscopic nephron preservation surgery: After general anesthesia was achieved, catheter was indwelled, lateral decubitus position was taken, and routine disinfection was performed. A 1.5-cm incision was made at the plain umbilicus of the outer edge of the rectus abdominis muscle on the affected side. The abdominal cavity was punctured with pneumoperitoneum needle, CO_2_ was injected to establish pneumoperitoneum, and 10-mmTrocar was inserted to connect the pneumoperitoneum. A trocar of 5 mm, 10 mm, and 5 mm was inserted about 2 cm below the border between the anterior axillar y line and the costal margin, about 3 cm below the midclavicular line and the border between the costal margin, and under the xiphoid process. Implantation of the operating instrument: First, the adhesion area between the affected intestinal tube and the abdominal wall with an electric hook was separated, then the lateral peritoneum in the right paracolic groove was opened, the ascending colon and colon liver area were fully dissociated, the left colon was pushed, the liver was stirred up, and the kidney was fully exposed. The perirenal fascia was opened in the middle of the kidney with an ultrasonic knife to separate perirenal fat, the tumor and the surrounding normal kidney tissue were fully exposed, then the pedicle vessels of the kidney were separated, and the renal artery was carefully separated to block the renal blood supply. After the dilated balloon catheter blocked the renal artery, the right kidney was continuously perfused with Ringer liquid at 4°C along the catheter, and the tumor was completely removed about 0.5 cm along the tumor margin. The wound bleeding points were sutured with 3-0 absorbable line, and the kidney wound was sutured with 2-0 absorbable line. The catheter balloon was released, and the blood supply to the kidney was restored. Because of the large wound, there is still a small amount of blood oozing, and the application of fiber compression has a good effect. After the specimen was removed and hemostasis was improved, one retroperitoneal drainage tube was indwelled, and the surgical incision was closed layer by layer. After the operation, the renal artery was blocked for 20 min during the operation. The patient returned to the ward safely, and the specimens were sent to the pathology after being examined by his family members.
3. Efficacy evaluation: Serum creatinine was reviewed at 1, 3, and 7 days after surgery, and renal GFR was reviewed at 6 months after surgery. Enhanced abdominal CT was reviewed at 1, 3, 6, and 12 months after surgery and once a year after surgery to confirm tumor recurrence. Postoperative imaging review was shown in **Figure 1** (picture of only one typical case was taken). Patients with renal insufficiency were followed up using enhanced MRI. Surgical success was defined as complete resection of the tumor during the operation, no significant tumor lesion was found in imaging examination 3 months after the operation, and no surgical method was changed. Success rate of operation = number of successful cases/number of surgical cases × 100%.

### Statistical analysis

SPSS version 24.0 was used for data analysis. Paired t- test was used to compare data before and after surgery, and P < 0.05 was considered statistically significant.

## Result

Eight of the nine patients were successfully completed without conversion to open or total nephrectomy. All the large postoperative specimens showed RAML, and no significant tumor recurrence was observed after 12 months of postoperative follow-up. The operative time (including renal artery catheterization time) was 105–260 min, with an average of 184 min. Only one patient underwent laparoscopic partial nephrectomy with renal artery occlusion for renal blood supply due to intolerance to renal artery inter ventional catheterization, and the renal artery occlusion time was 18–30 min, with an average of 23 min. Only the patients with transarterial clamp had more bleeding, and the other patients had no obvious bleeding. There was no significant difference in creatinine between preoperative and postoperative 7 days [(57.0 ± 12.35) µmol/L vs. (57.0 ± 9.03) µmol/L, P > 0.05]. GFR reexamination 6 months after surgery showed no significant changes in the kidney on the affected side compared with that before surgery [(47.37 ± 9.20) mL/min vs. (42.75 ± 8.77) mL/min, P > 0.05]. During the operation, only the patients with transarterial clamp had more bleeding, and no significant complications were observed in the remaining patients. The postoperative follow-up was more than 12 months. No further renal injury and tumor recurrence were found.

## Discussion

Previous studies have shown that laparoscopic partial nephrectomy can effectively reduce the recurrence rate of RAML. However, because kidney cells are very sensitive to heat ischemia (9, 10), during partial nephrectomy, blocking the renal arterial blood supply is an indispensable step to ensure clear field of vision in the operative area and reduce blood loss. A large number of studies have shown that tissue cells can effectively reduce metabolism at low temperature, so that they can maintain longer activity. Cryogenic perfusion has also been widely used in organ transplantation. Studies have shown that kidney hypothermia can effectively reduce kidney damage during kidney preservation surgery. Renal artery cold perfusion is a common physiological form of kidney hypothermia treatment (11–13), and its advantages lie in the following: ① rapid replacement of kidney residual blood to achieve kidney cooling; ② prevention of blood clotting in blood vessels; and ③ keeping the field clear. Ringer’s solution is selected because it is widely used to regulate water and electrolyte balance, which has better safety compared with normal saline (14). Therefore, laparoscopic partial nephrectomy assisted by renal artery cold perfusion is a relatively safe method. Laparoscopic partial nephrectomy assisted by renal artery cold perfusion has many advantages: (1) It reduced renal heat ischemia time. After low temperature perfusion, the consumption of oxygen and ATP of kidney cells is reduced, thus effectively prolonging the operation time, protecting renal function and greatly reducing the probability of postoperative renal function injury. (2) It provides a better intraoperative vision: Because of the vascular property of renal hamartoma, the tumor can be better distinguished from normal renal tissue after balloon dilation of renal artery and Ringer’s fluid perfusion at low temperature, thus facilitating surgical resection. In addition, because the residual blood of kidney is replaced by Ringer’s solution, the obstruction of blood to operation field can be reduced. (3) It reduces the damage to kidney blood vessels and saves the operation time. In previous partial nephrectomy, renal vessels are often clamped by arterial clamp, which will cause some damage to renal vessels, and intraoperative separation of renal portal vessels takes a lot of time. Renal artery balloon dilated by water injection *in vitro* can effectively block the renal artery blood supply, marked by the kidney parenchyma from red to white. (4) It avoids the loss of artery clip and improves the safety of surgery. Renal artery balloon occlusion of renal blood supply avoids the risk of hemorrhagic shock caused by the loss of the artery clip in previous surgical procedures and is more selective. If the tumor is supplied by a single vessel, then it can be placed in the donor artery.

## Conclusion

In our study, eight patients were successfully implanted with balloon catheters without significant intraoperative catheter displacement, bleeding, and other complications; no significant abnormalities in preoperative and postoperative renal function; and no long-term renal function abnormalities. Some previous studies have shown that rapid tumor nucleation and renal parenchyma preservation are effective methods to reduce near - and long-term renal injury (14–16). This surgical method reduces the time of hot ischemia and prolongs the time of renal ischemia, thus protecting renal function to a great extent. Therefore, these results indicate that this surgical procedure can be used as a treatment for renal hamartoma.

However, the new procedure has some limitations. First, compared with traditional laparoscopic partial nephrectomy, renal artery balloon placement is performed more often, which means prolonged operation time and increased surgical costs for patients. In addition, this study shows that the surgical procedure must be performed in a medical institution with an interventional radiology center. Second, this is a retrospective study with a small sample size, so the current results should be interpreted with caution. Further randomized controlled trials are needed to explore the safety and feasibility of the surgical method in the study.

## Data availability statement

The original contributions presented in the study are included in the article/supplementary material. Further inquiries can be directed to the corresponding author.

## Ethics statement

All study participants were informed about the planned procedure and signed informed consent.

## Author contributions

TY, SZ, and YM: Project development. CZ and HY: Manuscript writing. CZ, MZ, and ZD: Data collection and analysis. All authors contributed to the article and approved the submitted version.
